# Lactam Triterpenoids from the Bark of *Toona sinensis*

**DOI:** 10.1007/s13659-016-0108-4

**Published:** 2016-10-18

**Authors:** Qian-Qian Meng, Xing-Rong Peng, Shuang-Yang Lu, Luo-Sheng Wan, Xia Wang, Jin-Run Dong, Rui Chu, Lin Zhou, Xiao-Nian Li, Ming-Hua Qiu

**Affiliations:** 1 State Key Laboratory of Phytochemistry and Plant Resources in West China, Kunming Institute of Botany, Chinese Academy of Sciences, Kunming, 650201 People’s Republic of China; 2 University of the Chinese Academy of Sciences, Beijing, 100049 People’s Republic of China; 3 Yunnan University of Traditional Chinese Medicine, Kunming, 650500 People’s Republic of China

**Keywords:** *Toona sinensis*, Limonoids, Lactam triterpenoids, Cytotoxicity

## Abstract

Three new limonoid-type triterpenoids, namely toonasins A–C (**1**–**3**) with a rare lactam E ring, along with six known compounds (**4–9**) were isolated from the barks of *Toona sinensis*. The structures of new compounds were elucidated by interpretation of spectroscopic data, and the relative configuration of compound **1** was further characterized by X-ray crystallographic analyses. The isolated compounds were evaluated for their cytotoxic activities against five human tumor cell lines (HL-60, SMMC-7721, A-549, MCF-7 and SW480), and compounds **3** and** 5 **showed weak cytotoxicities.

**Electronic supplementary material:**

The online version of this article (doi:10.1007/s13659-016-0108-4) contains supplementary material, which is available to authorized users.

## Introduction

*Toona sinensis* is a shrub of Meliaceae distributed widely in Asian countries [1]. The leaves of *T. sinensis*, which contain a distinct flavor, are very popular in vegetarian cuisine and have long been used as a nutritious food in China and Malaysia [2]. In folk, almost every part of *T. sinensis*, including seeds, bark, root bark, petioles, and leaves, can be used to treat cold, rheumatic pain, stomach pain, and diarrhea without any irreversible side effects [3, 4]. Modern pharmacological researches also demonstrated that this plant showed wide spectrum of biological activities, such as antioxidant [5], anti-diabetes [6], anti-inflammatory [7], antimicrobial [8], antinociceptive [9] and anti-tumor [10], due to its plentiful chemical constituents (limonoids, flavonoids, phytols, coumarins and norcyteine derivatives) [11–14].

**Fig. 1:**
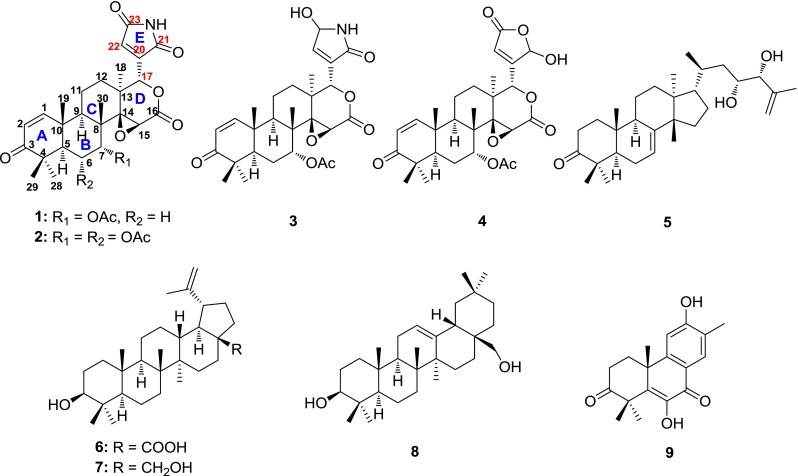
Structures of compounds **1**–**9** isolated from the bark of *Toona sinensis*

Limonoids were mainly identified from Meliaceae species and possessed fascinating structures [15] and various bioactivities [16–19]. Our previous phytochemical investigation of the Meliaceae species (*T. ciliata* and *Swietenia mahagoni*) led to the isolation of structurally diverse limonoids with anti-cancer and anti-bacterial effects [15, 20, 21]. In our continuing search for structurally interesting and biologically important chemical constituents, the bark of the title plant was investigated and three new limonoids, namely toonasins A–C (**1**–**3**), along with six known compounds, including one limonoid, photogedunin (**4)** [22], one tirucallane triterpenoid, bourjotinolone B (**5**) [23], three pentacyclic triterpenoids, betulinic acid (**6**) [24], betulin (**7**) [24], and erythrodiol (**8**) [25], as well as one diterpenoid, gossweilone (**9**) [26] (Fig. 1) were isolated. Among them, compounds **1**–**3** had a unique lactam E ring moiety and were first isolated from this species. The structures of new isolates were elucidated on the basis of the 1D, 2D NMR, and MS spectra. The structure of **1** was further confirmed by the X-ray crystallographic analyses. Subsequently, their cytotoxic activities were evaluated by MTT method.

## Results and Discussion

**Fig. 2:**
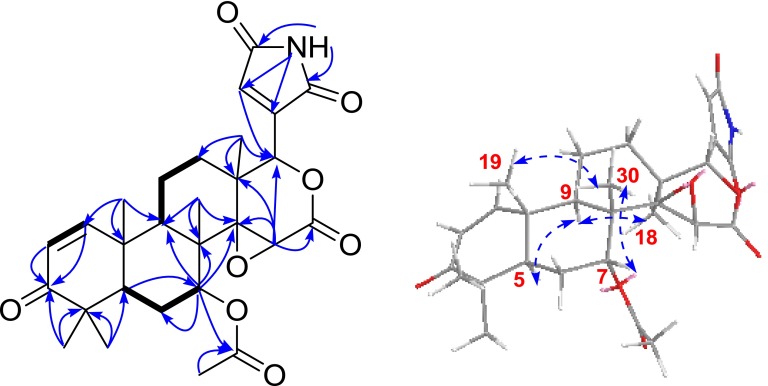
Key HMBC (H  C), ^1^H-^1^H COSY () and ROESY () correlations of **1**

Compound **1** was assigned a molecular formula of C_28_H_33_NO_8_ by HRESIMS and 1D NMR spectroscopic data (Table 1), which required 13 degrees of unsaturation. The IR spectrum revealed the presence of NH (3485 cm^−1^), carbonyl (1721 cm^−1^) and amide (1651 cm^−1^) groups. The ^1^H NMR spectrum of compound **1** showed five singlet methyls (*δ*_H_ 1.07, 1.08, 1.18, 1.23 and 1.26), a typical singlet methyl signal at *δ*_H_ 2.11 for acetyl, two oxymethines (*δ*_H_ 4.55, br s, H-7; *δ*_H_ 3.53, s, H-15), and three aromatic/olefinic methines (*δ*_H_ 7.12, d, *J* = 10.2 Hz, H-1; *δ*_H_ 5.90, d, *J* = 10.2 Hz, H-2; *δ*_H_ 6.72, s, H-22). The ^13^C-DEPT spectra of **1** displayed twenty-eight carbon resonances. Apart from an acetyl group, the remaining signals were assigned as five methyls, three methylenes, an *α*, *β*-unsaturated carbonyl (*δ*_C_ 157.0, C-1; *δ*_C_ 126.2, C-2; *δ*_C_ 204.2, C-3), a *δ*-lactone ring with a 14,15-epoxy fraction (*δ*_C_ 69.7, C-14; *δ*_C_ 56.8, C-15; *δ*_C_ 166.7, C-16; *δ*_C_ 75.4, C-17), and two lactam carbonyls (*δ*_C_ 169.6, C-21; *δ*_C_ 168.8, C-23), which were further supported by its HSQC, HMBC and ^1^H-^1^H COSY experiments (Fig. 2). Meanwhile, the HMBC correlations of H-7 to the acetyl, C-5, C-6, C-8, C-9, and C-14, and of H-5 and H-6 with C-7, along with the ^1^H-^1^H COSY correlations of H-5/H-6/H-7 illustrated that the acetoxyl was located at C-7. Above information suggested that **1** was a limonoid-type triterpenoid and resembled photogedunin [22] except that they had a different substituent at C-17.

**Table 1:** ^1^H (600 MHz) and ^13^C NMR (150 MHz) data of compounds **1**–**3** in CDCl_3_

Position	**1**		**2**		**3**	
	*δ*_C_	*δ*_H_ (*J* in Hz)	*δ*_C_	*δ*_H_ (*J* in Hz)	*δ*_C_	*δ*_H_ (*J* in Hz)
1	157.0, CH	7.12, d (10.2)	155.9, CH	7.08, d (10.1)	157.3, CH	7.13, d (10.2)
2	126.2, CH	5.90, d (10.2)	126.7, CH	5.97, d (10.0)	126.1, CH	5.88, d (10.1)
3	204.2, C		204.0, C		204.3, C	
4	44.2, C		44.9, C		44.2, C	
5	46.1, CH	2.17, dd (14.0, 2.7)	47.7, CH	2.52, d (12.4)	46.1, CH	2.17, d (12.4)
6	23.3, CH_2_	1.93, m	69.5, CH	5.27, dd (1.9, 12.4)	23.3, CH_2_	1.81, t (14.1) 1.93, m
7	73.3, CH	4.55, br s	72.5, CH	4.87, s	73.3, CH	4,53, br s
8	42.8, C		43.0, C		42.7, C	
9	39.4, CH	2.45, dd (12.9, 5.8)	38.1, CH	2.47, dd (12.9, 5.9)	39.6, CH	2.45, dd (12.8, 6.0)
10	40.3, C		40.5, C		40.2, C	
11	15.0, CH_2_	2.01, m 1.88, m	14.8, CH_2_	1.86, m 1.99, m	15.0, CH_2_	1.86, m 1.97, m
12	25.8, CH_2_	1.41, m 2.03, m	25.5, CH_2_	1.44, m 2.03, s	25.5, CH_2_	1.47, m 2.03, m
13	39.8, C		39.7, C		39.4, C	
14	69.7, C		69.6, C		69.8, C	
15	56.8, CH	3.53, s	55.9, CH	3.62, s	56.9, CH	3.50, s
16	166.7, C		166.7, C		167.5, C	
17	75.4, CH	5.68, s	75.1, CH	5.67, s	76.0, CH	5.57, s
18	17.6, CH_3_	1.26, s	17.6, CH_3_	1.25, s	18.5, CH_3_	1.16, s
19	19.9, CH_3_	1.23, s	21.5, CH_3_	1.22, s	19.9, CH_3_	1.23, s
20	145.0, C		144.8, C		136.6, C	
21	169.6, C		170.1, C		170.2, C	
22	133.2, CH	6.72, s	133.1, CH	6.72, s	146.3, CH	7.03, s
23	168.8, C		170.0, C		78.5, CH	5.63, d (7.02)
28	27.3, CH_3_	1.07, s	31.6, CH_3_	1.26, s	27.3, CH_3_	1.07, s
29	21.3, CH_3_	1.08, s	20.3, CH_3_	1.17, s	21.4, CH_3_	1.08, s
30	18.5, CH_3_	1.18, s	18.2, CH_3_	1.29, s	17.4, CH_3_	1.25, s
NH		7.84, s				6.35, s
6-COCH_3_			170.1, C			
6-COCH_3_			21.2, CH_3_	2.03, s		
7-COCH_3_	170.0, C		170.0, C		170.1, C	
7-COCH_3_	21.2, CH_3_	2.11, s	21.0, CH_3_	2.16, s	21.3, CH_3_	2.10, s

Assignments are supported with COSY, HSQC, and HMBC experiments

**Fig. 3:**
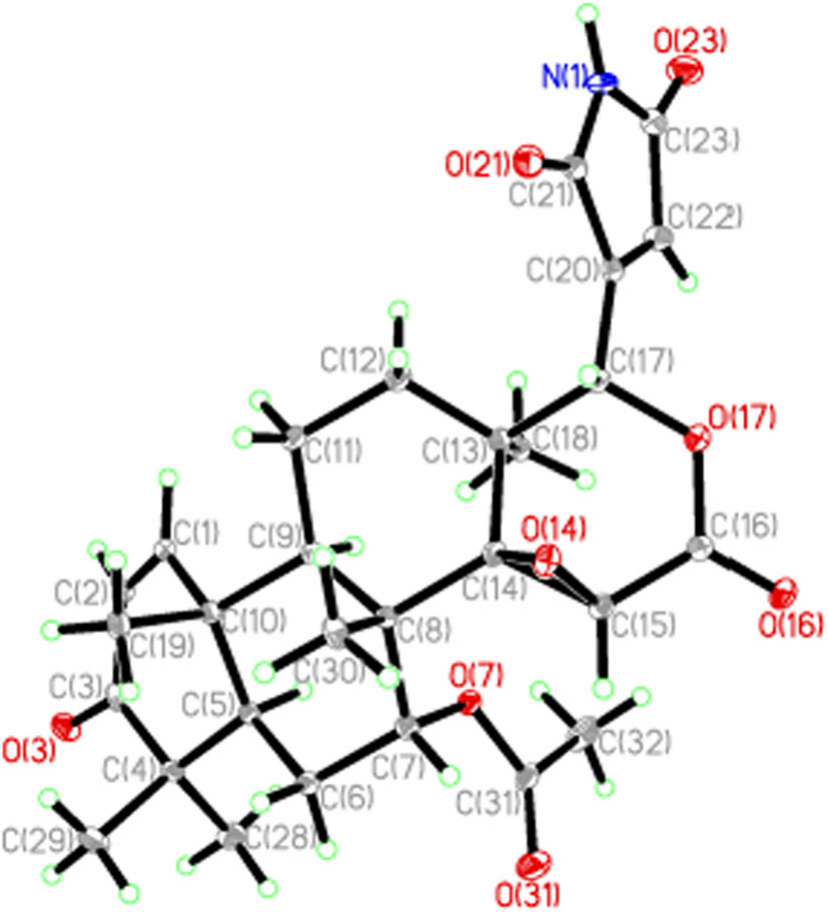
X-ray structure of **1**

The presence of a maleimide moiety at C-17 in **1** was established by the HMBC correlations of NH (*δ*_H_ 7.83, s) to C-20, C-21, C-22, and C-23, of H-22 (*δ*_H_ 6.72, s) to C-17, C-20, C-21, and C-23 (Fig. 2). The observed ROESY correlations (Fig. 2) of H-7/H_3_-19/H_3_-30, and of H-9/H_3_-18 allowed the assignment of 7-OAc and H-9 as *α*-oriented. To further confirm its skeleton of **1**, a single crystal was cultivated (Fig. 3). Based on above information, the structure of **1** was determined and named as toonasin A (**1**).

Compound **2** was obtained as a colorless needle with a molecular ion peak at *m/z* 592.2269 [M + Na]^+^ (calcd 569.2261) in the HRESIMS, coincided with the molecular formula of C_30_H_35_NO_10_. The IR spectrum exhibited absorption bands for NH (3435 cm^−1^), carbonyl (1745 cm^−1^) and amide (1657 cm^−1^) groups. Detailed comparison of the 1D NMR data between **1** and **2** (Table 1) showed that their main difference was an additional acetyl group [*δ*_*C*_ 170.1 (s), 21.2 (q)] in **2** instead of a methylene in **1**. Furthermore, the HMBC correlations of H-6 (*δ*_H_ 5.27, dd, *J* = 12.4 Hz) with the acetyl, C-5, C-7, and C-8, together with ^1^H-^1^H COSY correlations of H-5/H-6/H-7, indicated that the additional acetoxyl group was connected to C-6. Meanwhile, H-6 and H-7 showed the ROESY correlations with H_3_-18, suggesting that H-6 and H-7 was *β*-oriented. Finally, the structure of **2** was determined and named as toonasin B (**2**).

**Fig. 4:**
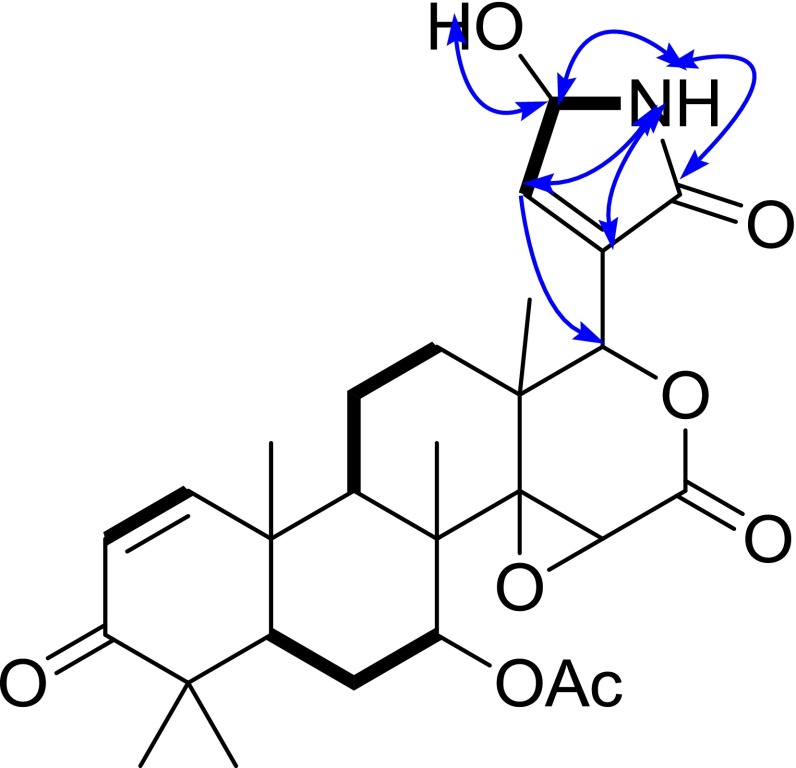
The selected HMBC (H  C), and ^1^H-^1^H COSY () correlations of compound **3**

Compound **3** displayed a molecule ion peak at *m/z* 536.2358 [M + Na]^+^ (calcd for C_28_H_35_ NO_8_, 513.2363) in the positive HRESIMS, consistent with a molecular formula of C_28_H_35_NO_8_. The IR absorption bands at 3438, 3328, 1637, and 1711 cm^−1^ indicated the presence of NH, OH, O=C–NH, and O=C. The 1D NMR spectroscopic data (Table 1) of **3** were similar with those of **1**, except for the presence of an oxymethine and the absence of a lactam carbonyl in **3**. Comparison of the 1D NMR data between **1** and **3** showed the highfield shift of C-20 (*δ*_C_ 145.0 for **1**; *δ*_C_ 136.6 for **3**) and the downfield shift of C-21 (*δ*_C_ 169.6 for **1**; *δ*_C_ 170.2 for **3**) and C-22 (*δ*_C_ 133.2 for **1**; *δ*_C_ 146.3 for **3**), suggesting that the carbonyl at C-23 in **1** could be replaced by the oxymethine (*δ*_C_ 78.5) in **3**. This deduction was further confirmed by the HMBC correlations of NH (*δ*_H_ 6.35, s) with C-20, C-21, C-22, and C-23, of H-23 with C-20, C-21, C-22, and C-17, together with the ^1^H-^1^H COSY correlations of NH/H-23/H-22 (Fig. 4). Hence, the structure of **3** was established and named as toonasin C (**3**).

Compounds **3**, **4**,** 5**,** 7 **and** 9** were evaluated for their cytotoxicities against five human tumor cell lines (HL-60, SMMC-7721, A-549, MCF-7 and SW480). The results (Table 2) showed that toonasin C (**3**) and bourjotinolone B (**5**) have weak inhibition activities against above cell lines with IC_50_ values of 12.00–25.00 μM.

**Table 2:** Cytotoxicity of compounds **3** and **5** (IC_50_: μM)

Compound	**HL-60**	**SMMC-7721**	**A-549**	**MCF-7**	**SW480**
**3**	18.61 ± 0.14	19.55 ± 0.19	15.07 ± 0.13	17.79 ± 0.15	12.47 ± 0.11
**5**	14.21 ± 0.12	18.14 ± 0.14	17.23 ± 0.14	24.78 ± 0.22	24.19 ± 0.22
**Cis-platin (MW300)**	2.03 ± 0.01	13.54 ± 0.12	12.56 ± 0.10	18.65 ± 0.15	19.70 ± 0.20

To the best our knowledge, the spire leaves of *T. sinensis* were used for a long time as health food and traditional medicine to treat rheumatoid arthritis, cevicitis, ruethritis, gastric ulcers, enteritis, and cancer [1]. However, phytochemical investigation mainly focused on flavonoids and polyphenols [28–30]. Pharmacological studies documented that the different parts of *T. sinensis*, including bark, seed, flower and root barks, also had various bioactivities [3, 4]. Meanwhile, previous research on the stem bark and leaves of this plant resulted in the isolation of a series of limonoids and the part of them exhibited significant cytotoxic potential [10, 31]. In the present paper, three novel limonoids with a unique lactam E ring were identified from the bark of *T. sinensis*, and toonasin C (**3**) showed comparable cytotoxic effect, with the positive control (Cis-platin), which indicated that limonoids from this plant should be paid more attention in order to explore and develop *T. sinensis* in more depth.

## Experimental Section

### General

The optical rotations were taken on a JASCO P-1020 polarimeter. UV spectra were record using a Shimadzu UV2401PC spectrophotometres. ^1^H and ^13^C NMR spectra were measured on Bruker AV-600 and DRX-500 instruments (Bruker, Zurich, Switzerland) using TMS as internal standard. Chemical shifts (*δ*) were expressed in *ppm* with reference to the TMS resonance. ESIMS and HRTOF-ESIMS data were recorded on an API QSTAR Pulsar spectrometer. EIMS and HRTOF-EIMS data were acquired on an Waters Auto Spec Premierp 776 spectrometer (America, Waters). Infrared spectra were recorded on a Bruker Tensor-27 instrument by using KBr pellets. An agilent 1100 series instrument equipped with Agilent ZORBAX SB-C18 column (5 μm, 4.6 mm × 250 mm) was used for high-performance liquid chromatography (HPLC) analysis. TLC was performed on precoated TLC plates (200–250 μM thickness, F254 Si gel 60, Qingdao Marine Chemical, Inc.) with compounds visualized by spraying the dried plates with 10 % aqueous H_2_SO_4_ followed by heating until dryness. Silica gel (200–300) mesh, Qingdao Marine Chemical, Inc.), Lichroprep RP-18 (40–63 μm, Fuji) and Sephadex LH-20 (20–150 μm, Pharmacia) were used for column chromatography.

The crystal structure of **1** was solved by direct method SHELXS-97 (Sheldrich, G. M. University of Gottingen; Gottingen, Germany, 1997) and the full-maxtrix least-squares deposited in the Cambridge Crystallographic Data Centre. Copies of these data can be obtained free of charge on application to CCDC via the Internet at www.ccdc.cam.ac.uk/conts/retrieving.html (or from the Cambridge Crystallographic Data Center, 12 Union Road, Cambridge CB2 1EZ, U.K.; fax (+44) 1223-336-033; or e-mail: deposit @ccdc.cam.ac.uk).

### Plant Material

The barks of *T. sinensis* were purchased from Beijing, China in May 2011, and identified by Prof. Jian Lou. A voucher specimen has been deposited at the State Key Laboratory of Phytochemistry and Plant Resources in West China, Kunming Institute of Botany, Chinese Academy of Sciences.

### Extraction and Isolation

The air-dried, powdered the bark of *T. sinensis* (3.5 kg) were extracted with acetone (98 % acetone/water) for three times (four days at a time) at room temperature. The solution of extracts was concentrated in vacuum to afford dark gummy residues, which was extracted with petroleum ether, chloroform and *n*-buthanol, respectively. The chloroform extract (38 g) was separated on a silica gel chromatography column (CC) with a gradient of Petroleum ether–Acetone (20:1, 10:1, 8:1, 5:1, 2:1, 1:2) as elution. Then, Petroleum ether–Acetone (5:1) part was subjected to reverse silica gel CC, eluting with H_2_O–MeOH (45:55, 35:65, 15:85) to give three fractions (A_5_, B_5_, C_5_). Fraction A_5_ was purified by silica gel CC (CHCl_3_–MeOH = 10:1) to gain compound **4** (128 mg). Fraction C_5_ was subjected to silica gel CC and eluted with CHCl_3_–MeOH = 10:1 to give two subfractions. Moreover, each of subfractions was further purified by semi-preparative HPLC (100 % MeOH) to yield compounds **5** (3 mg, 6.8 min), **6** (12 mg,7.6 min), **7** (8 mg, 8.0 min) and **8** (7 mg, 9.6 min) respectively.

Petroleum ether:Acetone (1:2) part was subjected to reverse silica gel CC, eluting with H_2_O–MeOH (55:45 to 0:100), to give fractions A_1_, B_1_, C_1_, D_1_, and E_1_. Compound 9 (5 mg) was isolated from fraction A_1_ by preparative TLC (CHCl_3_–MeOH, 30:1). Fraction B_1_ was purified by preparative TLC (CHCl_3_–MeOH, 20:1) to give compound** 3** (8 mg). Fraction C_1_ was subjected to silica gel CC (CHCl_3_–MeOH, 20:1) to give a pair of mixture, which was purified by P-TLC (CHCl_3_–MeOH, 30:1) to yield compounds **1** (30 mg) and **2** (3 mg).

#### Toonasin A (**1**)

Colorless needle; $\left[ \alpha \right]_{\text{D}}^{23}$ + 76.9°(*c* 0.1, CHCl_3_); UV (CHCl_3_) λ_max_ (logε): 239 (2.24), 223 (1.46), 220 (1.46) nm; IR (KBr) *v*_max_: 3485, 2964, 1721, 1651, 1370, 1240, 1032, 826 cm^−1^; For ^1^H and ^13^C-DEPT NMR spectroscopic data, see Table 1; HRESIMS: *m/z* 534.2211 [M + Na]^+^ (calcd for C_28_H_33_ NO_8_, 511.2206).

#### Crystal Data of **1**

C_28_H_33_NO_8_, M = 306.47; The crystal was colorless and transparent columnar, space group P2_1_2_1_2_1_; *a* = 8.7643 (12) Å, *b* = 11.3899 (16) Å, *c* = 27.007 (4) Å, *α* = *β* = *γ* = 90°, *V* = 2695.9 (6) Å^3^, *Z* = 4, *d* = 1.374 g/cm^3^. A colorless cube of dimensions 0.03 × 0.16 × 0.40 mm^3^ was used for X-ray measurement on a Bruker APEX DUO diffraction instrument with monochromatic graphite. Mo Kα radiation. The distance between the crystal and CCD detector is 50 mm. Of the 26448 reflections that were collected, 6661 were unique, and observable points (|F|^2^ ≥ 2σ|F|^2^) 4978. The crystal structure of **1** reported here is deposited with the Cambridge Crystallographic Data Centre (deposition number: 871944).

#### Toonasin B (**2**)

Colorless needle; $\left[ \alpha \right]_{\text{D}}^{23}$ + 85.3°(*c* 0.1, CHCl_3_); UV (CHCl_3_) λ_max_ (logε): 239 (2.11), 227 (1.52), 204 (1.38) nm; IR (KBr) *v*_max_: 3435, 2959, 2926, 1745, 1657, 1367, 1233, 1030, 931,827 cm^−1^; HRESIMS: *m/z* 592.2269 [M + Na]^+^ (calcd for C_30_H_35_ NO_10_, 569.2261).

#### Toonasin C (**3**)

Colorless needle; $\left[ \alpha \right]_{\text{D}}^{23}$ + 24.5°(*c* 0.1, CHCl_3_); UV (CHCl_3_) λ_max_ (logε): 239.4 (2.01), 208 (1.16), 203 (1.13) nm; IR (KBr) *v*_max_: 3438, 3398, 2925, 1711,1637, 1268,1124, 1025, 576 cm^−1^; HRESIMS: *m/z* 536.2358 [M + Na]^+^ (calcd for C_28_H_35_ NO_8_, 513.2363).

### Cytotoxicity Assays

There are five cancer cell lines including MCF-7, SMMC7721, HL-60, SW480 and A549, which were obtained from Shanghai cell bank in China. Cells were cultured in DMEM medium (Hyclone, USA), supplemented with 10 % fetal bovine serum (Hyclone, USA), in 5 % CO_2_ at 37 °C. Cytotoxicity was measured by standard MTT assay [27]. After the treatment of samples and positive control, cell viability was detected and a cell growth curve was graphed. The IC_50_ values were derived from the mean OD values of the triplicate tests versus drug concentration curves and expressed as mean ± standard deviation.

## Electronic Supplementary Material

Below is the link to the electronic supplementary material.

Supplementary material 1 (DOCX 3415 kb)
