# Supplementary material for: Lactam Triterpenoids from the Bark of Toona sinensis
Source: Nat Prod Bioprospect. 2016 Oct 18;6(5):239–45. doi: 10.1007/s13659-016-0108-4 (PMC5080210; doi:10.1007/s13659-016-0108-4)
Supplement: Supplementary file 1 — Supplementary material 1 (DOCX 3415 kb) [file 13659_2016_108_MOESM1_ESM.docx]

**Lactam Triterpenoids from the Bark of *Toona sinensis***

**Qianqian Meng^a,b,+^, Xingrong Peng^a,c,+^, Shuangyang Lu^a,b^ , Luosheng Wan^a,c^, Xia Wang^a,b^, Jinrun Dong^a,b^, Rui Chu^a,b^, Lin Zhou^a,c^, Xiaonian Li^a,b^, Minghua Qiu^a,b ,c^***

^a^ State Key Laboratory of Phytochemistry and Plant Resources in West China, Kunming Institute of Botany, Chinese Academy of Sciences, Kunming 650201, People′s Republic of China

^b^ University of the Chinese Academy of Sciences, Beijing 100049, People′s Republic China

^c^ Yunnan University of Traditional Chinese Medicine, Kunming, 650500, P. R. China;

Corresponding author: Tel: +86-0871-65223327. Fax: +86-0871-65223325. E-mail: [mhchiu@mail.kib.ac.cn](mailto:mhchiu@mail.kib.ac.cn) (M.–H. Qiu).

+ These authors contributed equally to this work.

**Contents of Supporting Information**

- **Figure S1 ^1^H NMR (600 MHz, CDCl_3_) spectrum of toonasin A (1).**
- **Figure S2 ^13^C NMR spectrum (150 MHz, CDCl_3_) of toonasin A (1).**
- **Figure S3 HSQC spectrum of toonasin A (1).**
- **Figure S4 HMBC spectrum of toonasin A (1).**
- **Figure S5 ROESY spectrum of toonasin A (1).**
- **Figure S6 ^1^H-^1^H COSY spectra of toonasin A (1)**
- **Figure S7 ^1^H NMR (600 MHz, CDCl_3_) spectrum of toonasin B (2).**
- **Figure S8 ^13^C NMR spectrum (150 MHz, CDCl_3_) of toonasin B (2).**
- **Figure S9 HSQC spectrum of toonasin B (2).**
- **Figure S10 HMBC spectrum of toonasin B (2).**
- **Figure S11 ROESY spectrum of toonasin B (2).**
- **Figure S12 ^1^H-^1^H COSY spectra of toonasin B (2)**
- **Figure S13 ^1^H NMR (600 MHz, CDCl_3_) spectrum of toonasin C (3).**
- **Figure S14 ^13^C NMR spectrum (150 MHz, CDCl_3_) of toonasin C (3).**
- **Figure S15 HSQC spectrum of toonasin C (3).**
- **Figure S16 HMBC spectrum of toonasin C (3).**
- **Figure S17 ROESY spectrum of toonasin C (3).**
- **Figure S18 ^1^H-^1^H COSY spectra of toonasin C (3).**
- **Figure S19 Data of single-crystal X-ray diffraction of toonasin A (1).**

**Figure S1. ^1^H NMR** **(600 MHz, CDCl_3_) spectrum of toonasin A (1).**

**Figure S2. ^13^C NMR spectrum (150 MHz, CDCl_3_) of toonasin A (1).**

**
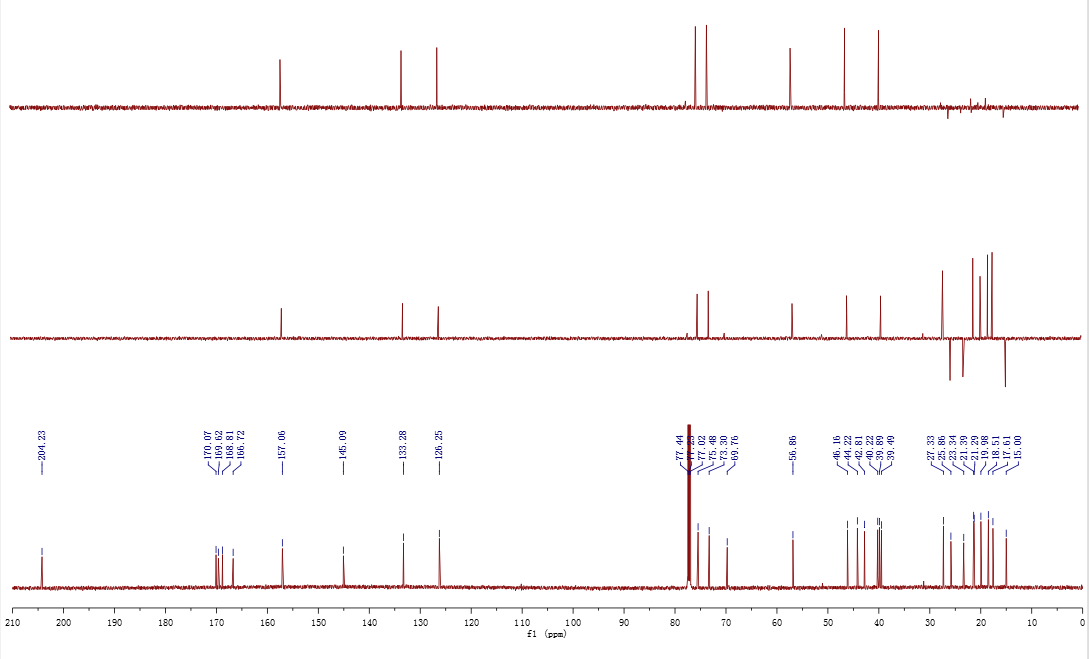
**

**Figure S3. HSQC spectrum of toonasin A (1).**

**
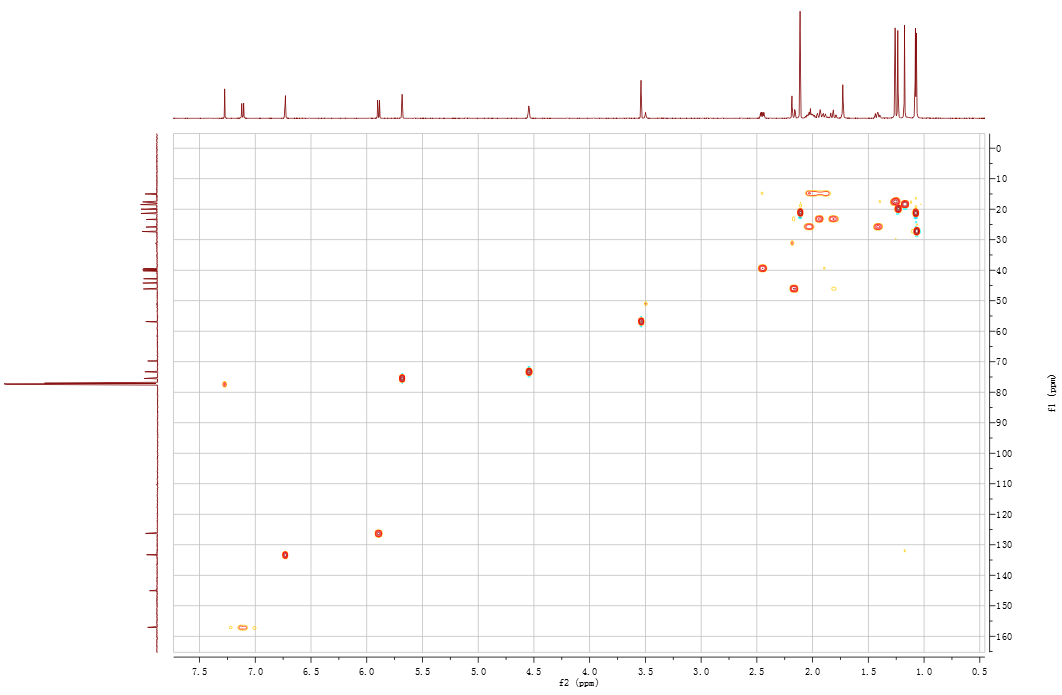
**

**Figure S4. ^1^**

**Figure S4. ^1^H-^1^H COSY spectrum of toonasin A (1) .**


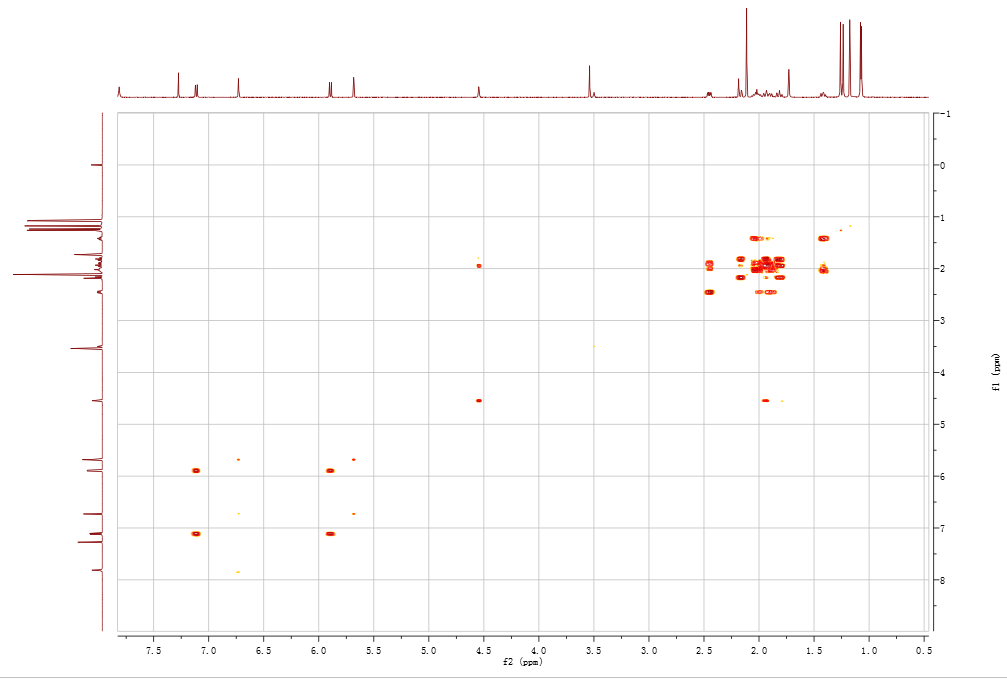


**Figure S5. HMBC spectrum of toonasin A (1).**


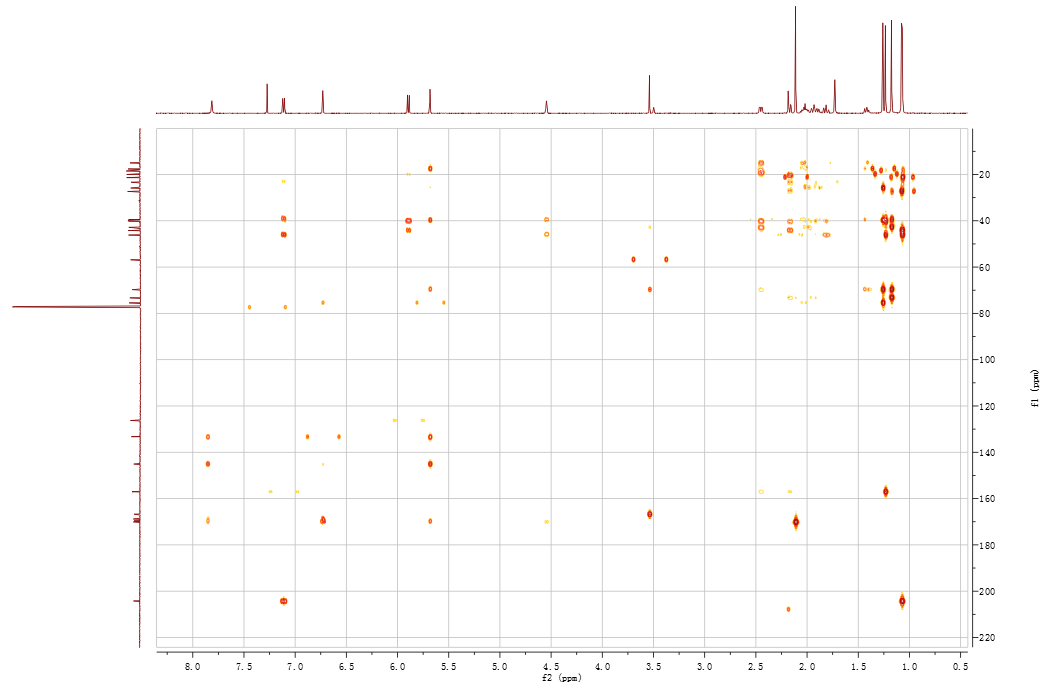


**Figure S6. The ROESY spectrum of toonasin A (1).**


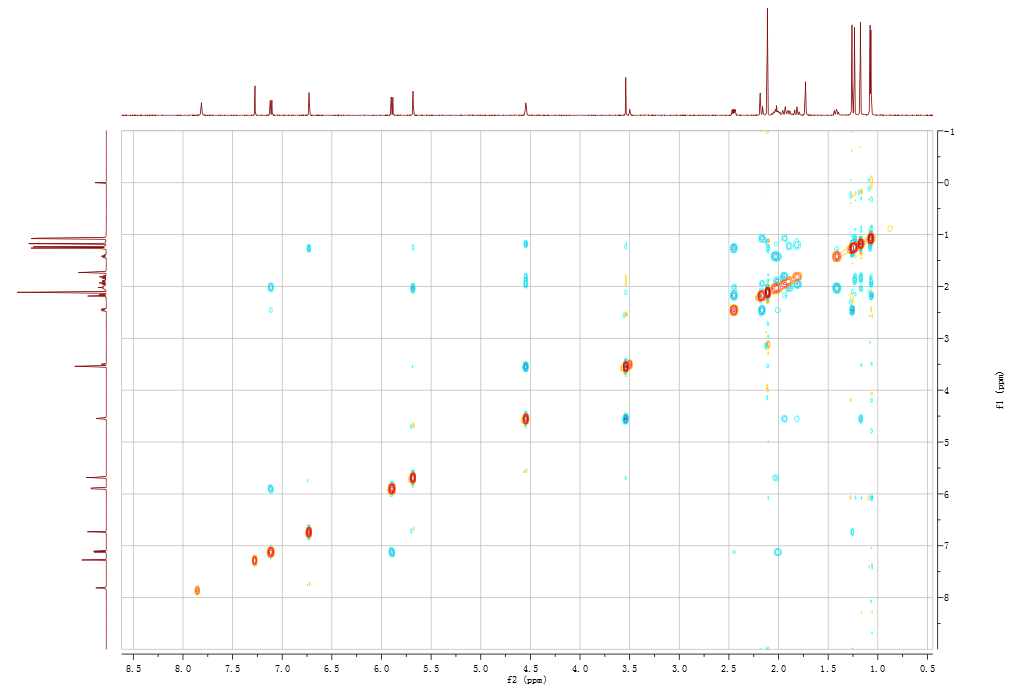


**Figure S7. ^1^H NMR (600 MHz, CDCl_3_) spectrum of toonasin B (2).**

**Figure S8. ^13^C NMR spectrum (150 MHz, CDCl_3_) of toonasin B (2).**

**
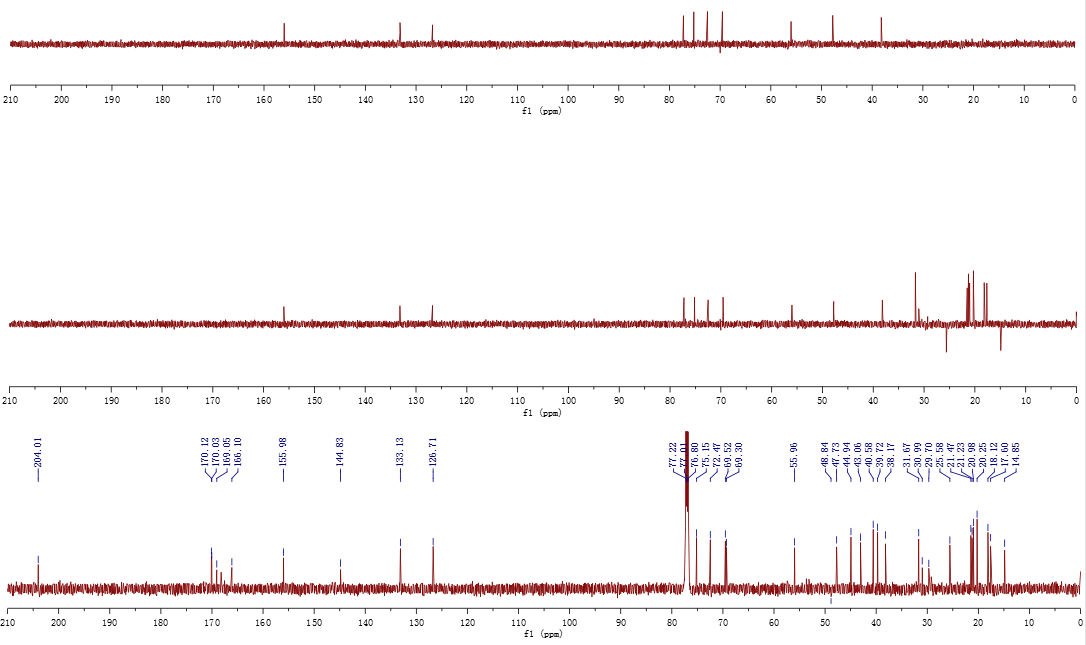
**

**Figure S9. HSQC spectrum of toonasin B (2).**

**
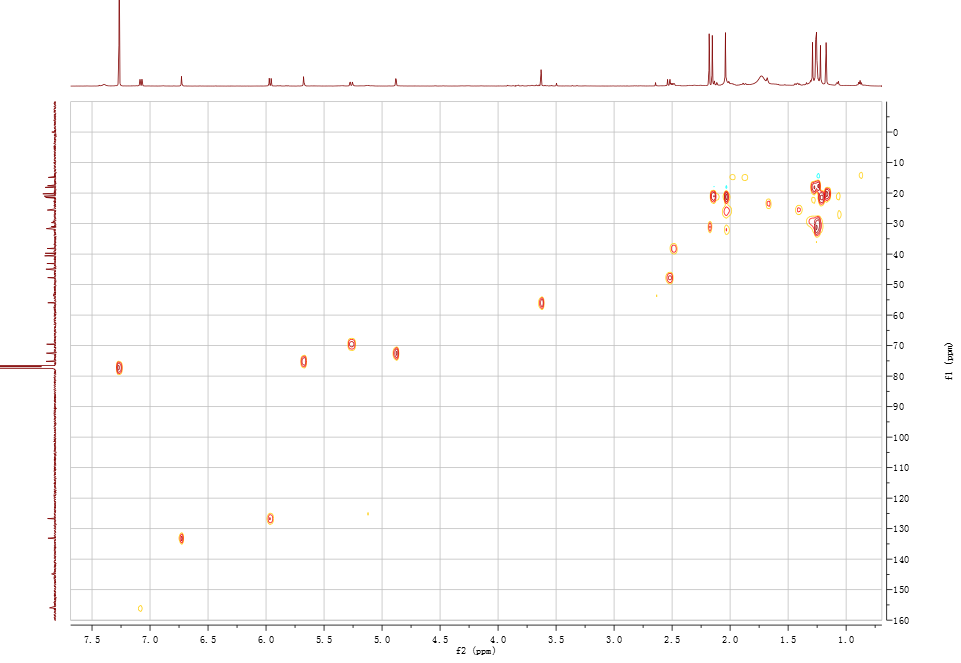
**

**Figure S10. ^1^H-^1^H COSY spectrum of toonasin B (2) .**

**
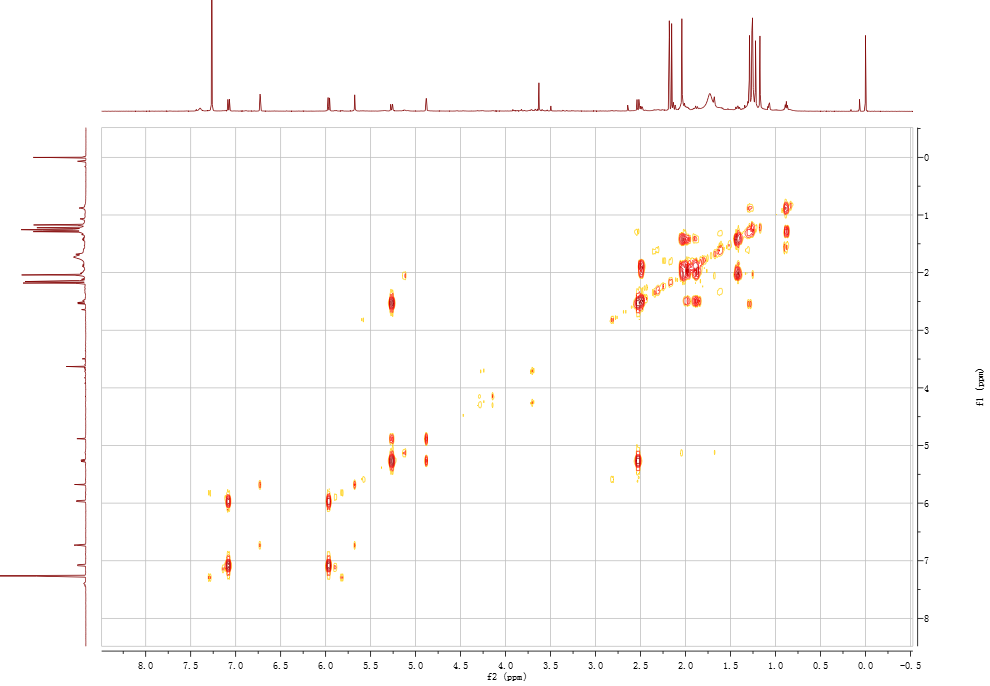
**

**Figure S11. HMBC spectrum of toonasin B (2).**

**
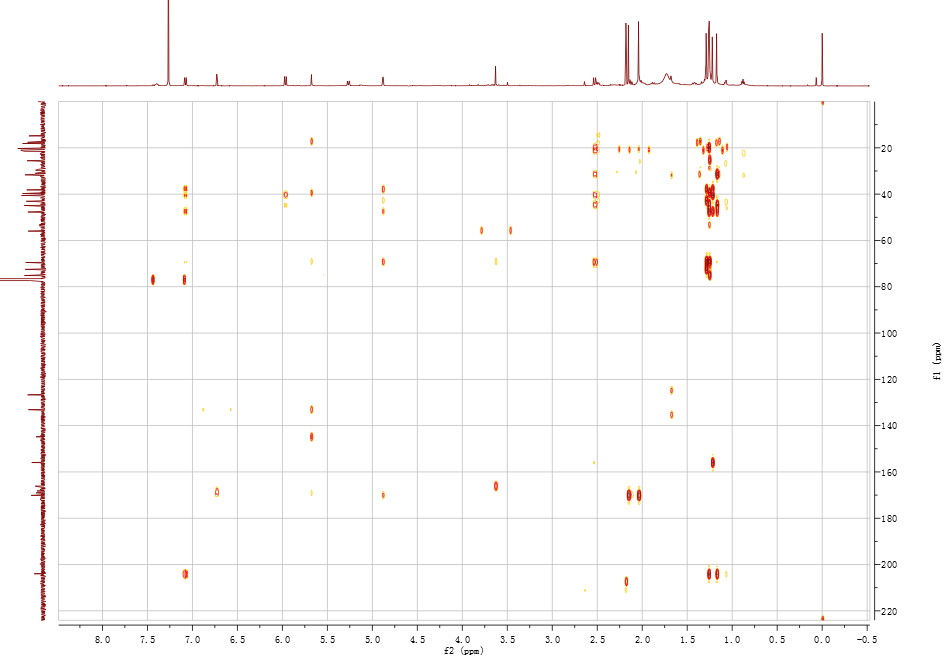
**

**Figure S12. The ROESY spectrum of toonasin B (2).**

**
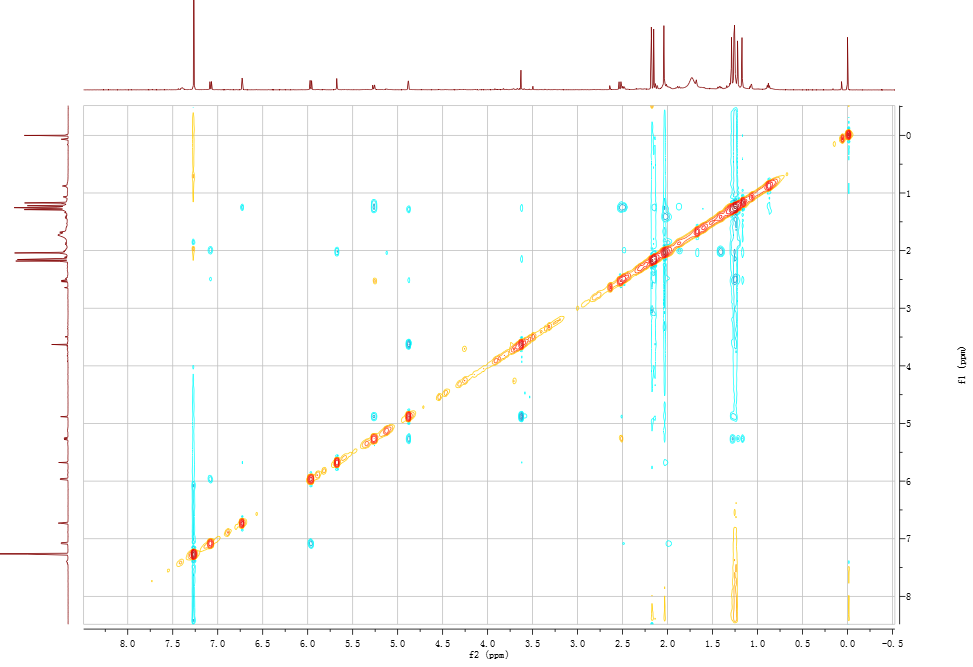
**

**Figure S13. ^1^H NMR (600 MHz, CDCl_3_) spectrum of toonasin C (3).**

**Figure S14. ^13^C NMR spectrum (150 MHz, CDCl_3_) of toonasin C (3).**


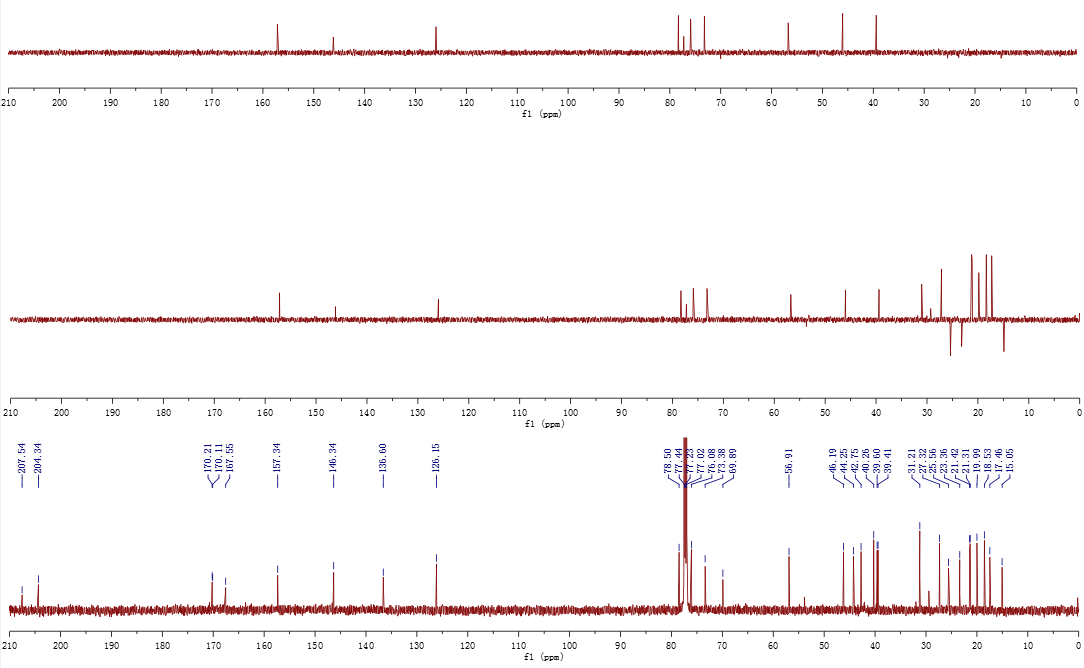

**Figure S15. HSQC spectrum of toonasin C (3).**

**
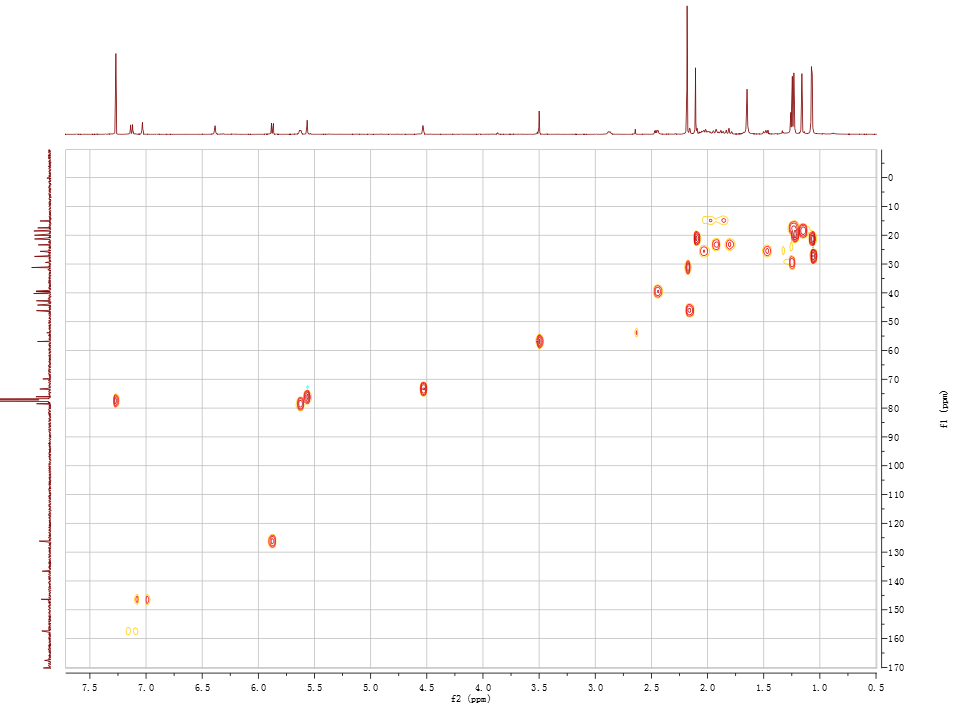
**

**Figure S16. ^1^H-^1^H COSY spectrum of toonasin C (3) .**

**
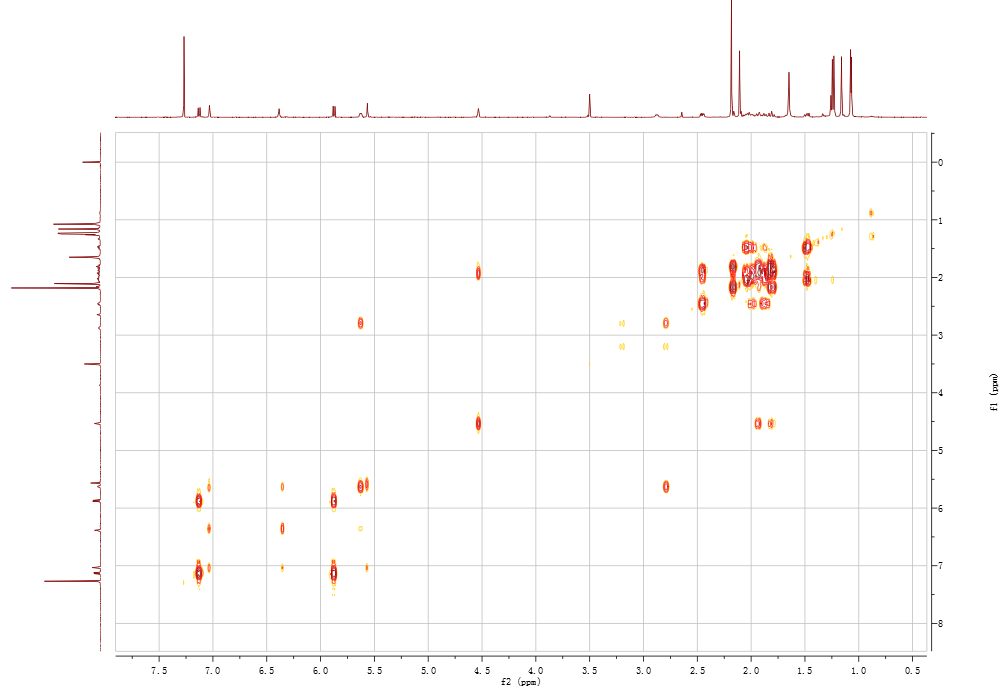
**

**Figure S17. HMBC spectrum of toonasin C (3).**

**
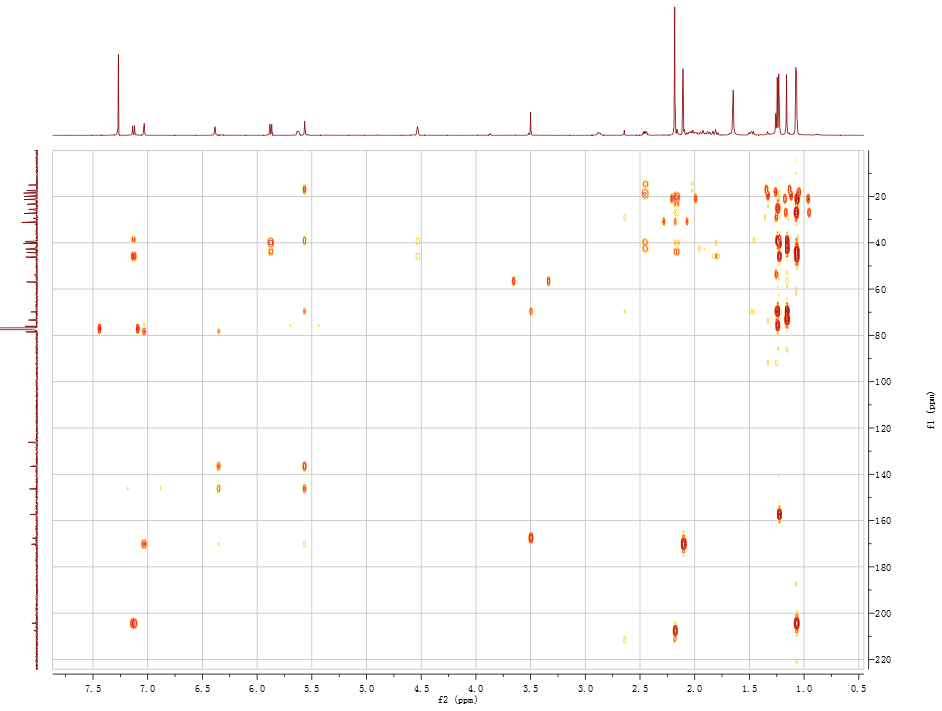
**

**Figure S18. The ROESY spectrum of toonasin C (3).**

**
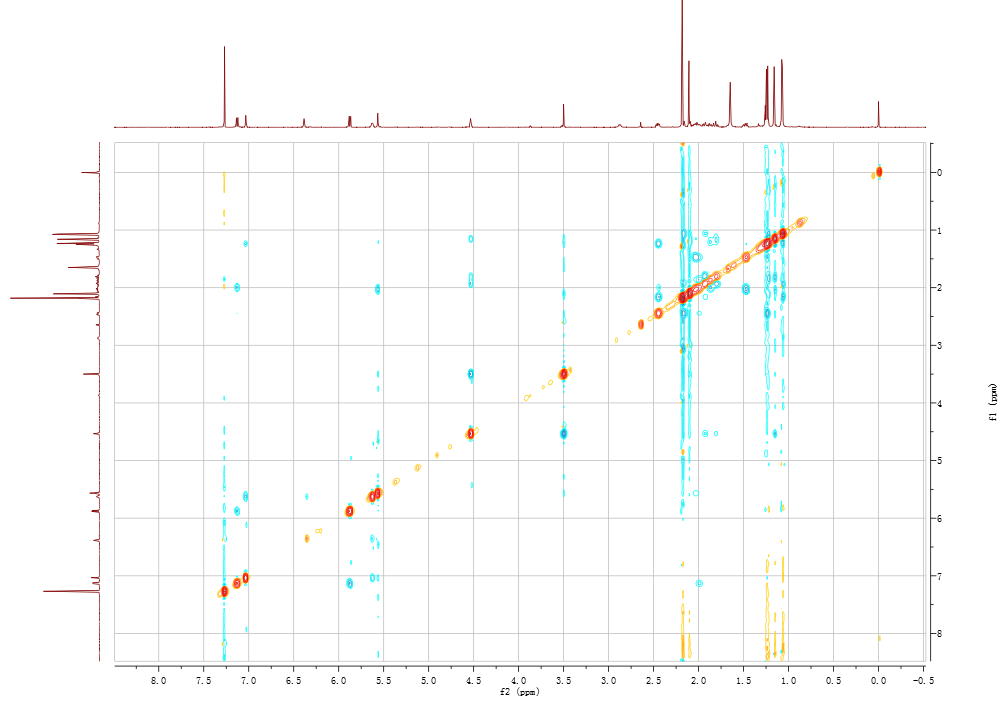
**

**Figure S19. Data of single-crystal X-ray diffraction of toonasin A (1)**

C_28_H_33_NO_8_, M = 306.47; The crystal was colorless and transparent columnar, space group P2_1_2_1_2_1_; *a* = 8.7643 (12) Å，*b* = 11.3899 (16) Å，*c* = 27.007 (4) Å, *α* = *β* = *γ* = 90°, *V* = 2695.9 (6) Å^3^, *Z* = 4, *d* = 1.374 g/cm^3^. A colorless cube of dimensions 0.03 × 0.16 × 0.40 mm^3^ was used for X-ray measurement on a Bruker APEX DUO diffraction instrument with monochromatic graphite. Mo Kα radiation. The distance between the crystal and CCD detector is 50 mm. Of the 26448 reflections that were collected, 6661 were unique, and observable points (|F|^2^ ≥ 2σ|F|^2^) 4978.

**
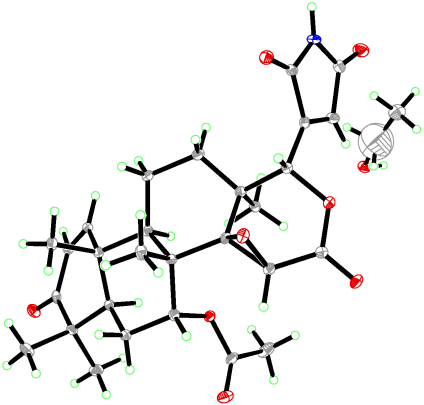
**

View of a molecule of fid25 with the atom-labelling scheme.

Displacement ellipsoids are drawn at the 30% probability level.

**
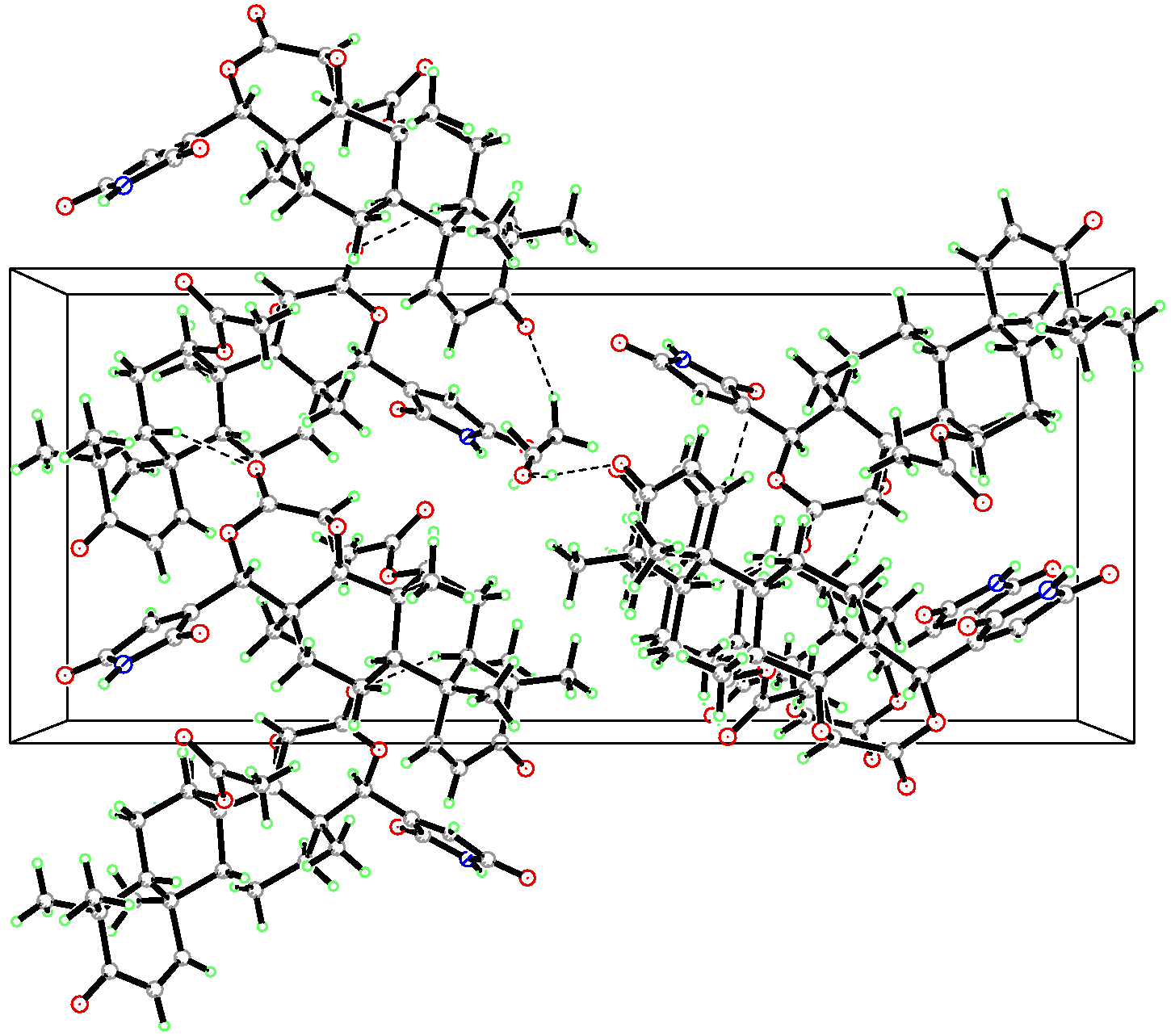
**

View of the hydrogen-bonded motif of fid25.

Hydrogen-bonds are shown as dashed lines.

**Table 1. Crystal data and structure refinement for toonasin A (1).** Identification code k20a

Empirical formula C_30_ H_39_ N O_9_

Formula weight 557.62

Temperature 100(2) K

Wavelength 0.71073 A

Crystal system, space group Orthorhombic, P 21 21 21

Unit cell dimensions a = 8.7643(12) A alpha = 90 deg.

b = 11.3899(16) A beta = 90 deg.

c = 27.007(4) A gamma = 90 deg.

Volume 2695.9(6) A^3

Z, Calculated density 4, 1.374 Mg/m^3

Absorption coefficient 0.101 mm^-1

F(000) 1192

Crystal size 0.40 x 0.16 x 0.03 mm

Theta range for data collection 1.51 to 28.24 deg.

Limiting indices -11<=h<=11, -15<=k<=15, -30<=l<=35

Reflections collected / unique 26448 / 6661 [R(int) = 0.0872]

Completeness to theta = 28.24 100.0 %

Absorption correction Semi-empirical from equivalents

Max. and min. transmission 0.9970 and 0.9607

Refinement method Full-matrix least-squares on F^2

Data / restraints / parameters 6661 / 0 / 369

Goodness-of-fit on F^2 1.098

Final R indices [I>2sigma(I)] R1 = 0.0573, wR2 = 0.1248

R indices (all data) R1 = 0.0899, wR2 = 0.1373

Absolute structure parameter 0.3(11)

Largest diff. peak and hole 0.448 and -0.522 e.A^-3
